# Reversible Total Vision Loss Caused by Severe Metformin-associated Lactic Acidosis: A Case Report

**DOI:** 10.5811/cpcem.2021.3.51141

**Published:** 2021-05-06

**Authors:** Andrew Koons, Alexandra M. Amaducci, Kenneth D. Katz

**Affiliations:** Lehigh Valley Health Network, Department of Emergency and Hospital Medicine, Allentown, Pennsylvania

**Keywords:** Metformin, metabolic, diabetic, vision loss, concentrations, case report

## Abstract

**Introduction:**

Metformin is a biguanide used to treat diabetes mellitus (DM). Metformin-associated lactic acidosis (MALA) carries a high mortality and can occur in patients with renal failure from drug bioaccumulation. Reversible vision loss is a highly unusual, rarely reported complication of MALA. We present a case of a patient whose serum metformin concentration was unusually high and associated with vision loss.

**Case Report:**

A 60-year-old woman presented to an outside hospital emergency department with acute vision loss after being found at home confused, somnolent, and hypoglycemic, having last being seen normal two days prior. She reported vomiting and diarrhea during that time and a recently treated urinary tract infection. The visual loss resolved with continuous renal replacement therapy.

**Conclusion:**

This novel case of a patient with Type II DM prescribed metformin and insulin who developed reversible vision loss while suffering from MALA highlights the potential for vision loss in association with MALA.

## INTRODUCTION

Metformin-associated lactic acidosis (MALA) is a potentially life-threatening adverse effect of metformin use with the incidence ranging from approximately 10–11 in 100,000 persons per year.^1^,^2^ Historically, from 1960–2000, it carried a 50% mortality rate; however, the mortality rate has improved to approximately 25–30% since 2000.^3^,^4^ Reversible vision loss is a highly unusual complication of MALA and has very rarely been reported.^5^–^9^

Metformin is a small drug (165 kilodalton) with an oral bioavailability of 50%. Since it is not metabolized by the liver, tubular secretion is the primary elimination route.^10^ The mechanism of action of metformin includes affecting the formation of adenosine triphosphate production by decreasing the efficiency of mitochondrial oxidative phosphorylation.^11^ Another mechanism of metformin includes the inhibition of mitochondrial glycerophosphate dehydrogenase. When this is affected, the balance of cytosolic nicotinamide adenine dinucleotide and hydrogen (NADH) is altered. There is then more NADH available, which decreases lactate to pyruvate conversion.^12^ Therefore, lactate can accrue, either as a result of increased metformin ingestion or decreased clearance.^13^ To maintain acid-base homeostasis, lactate is metabolized by gluconeogenesis or oxidation after converting to pyruvate, and the proton reacts with bicarbonate to form water and carbon dioxide.^13^ The kidney replenishes bicarbonate by reabsorption at the tubular level or net acid secretion.^14^,^15^ However, once these compensatory mechanisms are exhausted, bicarbonate is depleted, and lactate accumulates. Despite administering intravenous (IV) sodium bicarbonate to replace the depleted bicarbonate stores, the patient may require extracorporeal removal of these substances to aid in combating the effects of metformin by both removing metformin and correcting the acid-base disturbances.

## CASE REPORT

A 60-year-old woman with past medical history of diabetes mellitus prescribed insulin and metformin developed total vision loss. She was transported to the emergency department (ED) after being discovered at home confused, somnolent, and hypoglycemic, and last seen normal two days prior. The patient reported vomiting, diarrhea, and a recently treated urinary tract infection (UTI). She was never able to provide an exact history, but her sister reported to the ED staff that she had developed a UTI treated with an unknown antibiotic approximately one week prior to the current visit. The patient also reported to her sister diarrhea and weakness for one day prior to arrival to ED. Per emergency medical services (EMS), her initial blood glucose was 42 milligrams per deciliter (mg/dL) (reference range: 70–100 mg/dL) and improved after IV administration of 25 grams of glucose in a 50-cubic centimeter prefilled syringe (50% dextrose). No suspicion of surreptitious ingestion of medications was described by either EMS or family.

In the ED the patient’s vital signs were as follows: temperature 31.1°C (88°F); heart rate 58 beats per minute; respiratory rate 22 breaths per minute; blood pressure 90/43 millimeters mercury; and oxygen saturation 100% on room air. On physical examination, she was awake but ill-appearing and confused, demonstrating neither any vision perception nor blink reflex. Notable laboratory results are listed in the Table. Rewarming measures, IV fluids, and IV infusions of sodium bicarbonate, vasopressors (norepinephrine and vasopressin), folic acid, thiamine, 4-methylpyrazole, and empiric antibiotics (vancomycin and cefepime) were all administered. The patient received 4-methylpyrazole due to initial diagnostic uncertainty and significantly low bicarbonate concentration; it was discontinued when toxic alcohol ingestion was eliminated. The patient was moved to an intensive care unit (ICU) at a tertiary care referral center

**CPC-EM Capsule**

What do we already know about this clinical entity?

*Metformin Associated Lactic Acidosis (MALA) is a life-threatening adverse effect of metformin usage with a mortality rate of approximately 25–30%.*

What makes this presentation of disease reportable?

*This case presents a rare symptom of MALA, reversible vision loss.*

What is the major learning point?

*In order for emergency physicians to expedite proper critical care, recognizing the common and uncommon signs of MALA is crucial.*

How might this improve emergency medicine practice?

*By noticing the symptoms of metformin associated lactic acidosis, it will allow emergency physicians to provide best practice patient care.*

In the ICU the patient received continuous renal replacement therapy due to anuria. Continuous renal replacement therapy was initiated instead of hemodialysis due to persistent hypotension despite multiple vasopressors. She also was treated with IV sodium bicarbonate given her profound and persistent acidemia. Her vision improved, and she was able to track movement and perform extraocular movements. Eventually she had total resolution of her visual deficits. However, she developed pulmonary edema requiring intubation and, despite some improvement in acidemia and kidney function (Figure), she required maximal vasopressor support including IV infusions of epinephrine, norepinephrine, vasopressin, angiotensin, methylene blue, and steroids.

Admission and subsequent daily serum metformin concentrations measured 57 micrograms per milliliter (μg/mL), 42 μg/mL, and 13 μg/mL (therapeutic 1–2 μg/mL; MALA typically associated with more than 5 μg/mL). Serum liquid chromatography and mass spectroscopy toxicology screen did not include metformin concentrations and detected no other xenobiotics. Blood cultures, viral panels, chest radiograph, and chest, abdomen and pelvis computed tomography were unrevealing other than “minimal enterocolitis.” The patient unfortunately expired on hospital day three.

## DISCUSSION

Recommended indications (and associated levels of evidence) for extracorporeal treatment for metformin poisoning include the following: lactate concentration greater than 20 millimoles per liter on day one, pH less than or equal to 7.0 (day one), shock (day one), failure of standard supportive measures (day one), and decreased level of consciousness on day two.^4^

Documented vision loss associated with MALA and reversible blindness is very rare, and especially unique to this case is the highest recorded, concurrent metformin concentration (57 μg/mL). Kreshak et al reported a metformin concentration of 28 μg/mL in a patient with vision loss; the patient fully recovered after hemdialysis.^5^ Cigarran et al described a 54-year-old male patient with funduscopic examination findings revealing bilateral proliferative diabetic retinopathy with vitreous hemorrhage. This patient also had recovery of vision, but no serum concentration was available.^6^

The proposed mechanism of MALA-induced vision loss is that function of the retinal cells is related to the potential hydrogen (pH).^5^ Studies demonstrate in certain animal species such as fish, tiger, and salamanders that there is a pH-sensitive response to light by the retinal horizontal cells.^5^ These cells are laterally interconnecting neurons located in the inner nuclear layer of the retina that help integrate input from multiple photoreceptor cells. Animal models demonstrate that this transmission is disrupted in acidosis with a serum pH less than 7.0. If extrapolated to humans, this could explain the reason for loss of vision in the associated severe acidosis.^6^ Sudden vision loss has also been reported previously with both diabetic and alcoholic ketoacidosis. The common denominator among these patients was both a severe metabolic acidosis and reversal of abnormal vision symptoms with correction of serum pH.^7^ This patient had improvement and then resolution of her vision loss as her pH rose above 7.0. The patient’s serum metformin concentration of 57 μg/mL (therapeutic range 1–2 μg/mL) is the highest reported concentration associated with MALA-induced vision loss.

## CONCLUSION

This is a novel case of a patient with Type II diabetes mellitus prescribed metformin and insulin who developed MALA-induced, reversible vision loss with the highest measured, concurrent metformin concentration. Because MALA portends a high mortality, it is crucial for emergency physicians to recognize both the common and uncommon signs and symptoms to expedite proper critical care.

## Figures and Tables

**Figure:**
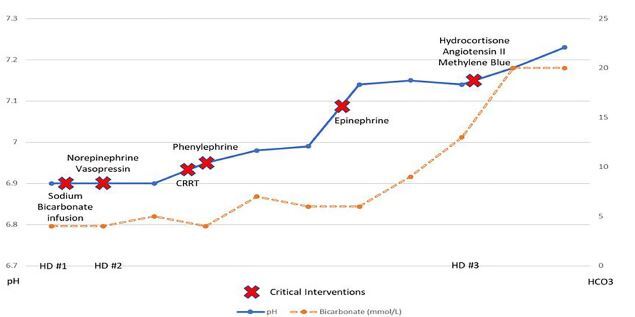
Laboratory findings of patient during hospital stay. *HD*, hospital days; *CCRT*, continuous renal replacement therapy; *HCO_3_*, bicarbonate.

**Table:** Notable lab results for patient presenting with total vision loss.

Test name	Result (reference range)
WBC	28,900 K/cmm (4.0–10.0 K/cmm)
Hgb	10.7 g/dL (11.5–14.5 g/dL),
Hct	36.6% (35.0–43.0%)
Platelet	261,000 (140–350 thou/cmm)
Glucose	216 mg/dL (70–100 mg/dL)
BUN	74 mg/dL (7–18 mg/dL)
Creatinine	9.38 mg/dL (0.40–1.00 mg/dL)
Na^+^	135 mmol/L (136–145 mmol/L)
K^+^	5.8 mmol/L (3.5–5.1 mmol/L)
Cl^−^	93 mmol/L (98–107 mmol/L)
HCO_3_^−^	4 mmol/L (21–32 mmol/L)
CPK	285 U/L (less than201 U/L)
Lipase	857 U/L (73–393 U/L)
Lactate	22.3 mmol/L (0.4–2.0 mmol/L)
Troponin I	0.06 ng/mL (0.00–0.05 ng/mL)
Pro-BNP	3912 pg/mL (0–125 pg/mL)
Prothrombin time	19.2 sec (12.0–14.6 sec)
Initial arterial blood gas on 4 liters nasal cannula	pH <6.94 (7.35–7.45), CO_2_ <20 mmol/L (32–45 mmol/L), pO_2_ 188 mm Hg (83–108 mm Hg)
Acetaminophen, salicylate, ethanol, and toxic alcohol panel	All negative

*WBC*, white blood cells; *K*, thousands; *cmm*, cubic millimeter; *Hgb*, hemoglobin; *g*, grams; *dL*, deciliter; *Hct*, hematocrit; *thou*, thousands; *mg*, milligrams; *BUN*, blood urea nitrogen; *Cr*, creatinine; *Na^+^*, sodium; *mmol*, millimoles per liter; *K^+^*, potassium; *Cl*^−^, chloride; *HCO_3_*^−^, bicarbonate; *CPK*, creatine phosphokinase; *U*, units; *ng*, nanograms; *mL*, milliliters; *pg*, picograms; *sec*, seconds; *CO_2_*, carbon dioxide; *pO_2_,* partial pressure of oxygen; *mm Hg*, millimeters of mercury.

## References

[1] Richy FF, Sabidó-Espin M, Guedes S (2014). Incidence of lactic acidosis in patients with type 2 diabetes with and without renal impairment treated with metformin: a retrospective cohort study. Diabetes Care.

[2] Li L, Jick S, Gopalakrishnan C (2017). Metformin use and risk of lactic acidosis in people with diabetes with and without renal impairment: a cohort study in Denmark and the UK. Diabet Med.

[3] Kajbaf F, Lalau JD (2013). The prognostic value of blood pH and lactate and metformin concentrations in severe metformin-associated lactic acidosis. BMC Pharmacol Toxicol.

[4] Calello DP, Liu KD, Wiegand TJ (2015). Extracorporeal treatment for metformin poisoning: systematic review and recommendations from the Extracorporeal Treatments in Poisoning Workgroup. Crit Care Med.

[5] Kreshak AA, Clark RF (2010). Transient vision loss in a patient with metformin-associated lactic acidosis. Am J Emerg Med.

[6] Cigarrán S, Rodriguez ML, Pousa M (2012). Transient vision loss in a patient with severe metformin-associated lactic acidosis. QJM.

[7] Hashmi M, Mahmood SN, Madhwani V (2018). Turning a blind eye: acute vision loss from an unexpected cause. Am J Respir Crit Care Med.

[8] Jeon JW, Choi W, Kim HR (2019). Transient blindness in a patient with severe metformin-associated lactic acidosis (MALA). Electrolyte Blood Press.

[9] Ryu S, Oh SK, Son SH (2019). Reversible acute blindness in suspected metformin-associated lactic acidosis. J Emerg Med.

[10] Peters N, Jay N, Barraud D (2008). Metformin-associated lactic acidosis in an intensive care unit. Crit Care.

[11] Andrzejewski S, Gravel SP, Pollak M (2014). Metformin directly acts on mitochondria to alter cellular bioenergetics. Cancer Metab.

[12] Madiraju AK, Erion DM, Rahimi Y (2014). Metformin suppresses gluconeogenesis by inhibiting mitochondrial glycerophosphate dehydrogenase. Nature.

[13] Yeh HC, Ting IW, Tsai CW (2017). Serum lactate level and mortality in metformin-associated lactic acidosis requiring renal replacement therapy: a systematic review of case reports and case series. BMC Nephrol.

[14] Abu Hossain S, Chaudhry FA, Zahedi K (2011). Cellular and molecular basis of increased ammoniagenesis in potassium deprivation. Am J Physiol Renal Physiol.

[15] Jacobs RA, Meinild AK, Nordsborg NB (2013). Lactate oxidation in human skeletal muscle mitochondria. Am J Physiol Endocrinol Metab.

